# Protein phosphatase 2A regulatory subunit B55α functions in mouse oocyte maturation and early embryonic development

**DOI:** 10.18632/oncotarget.15927

**Published:** 2017-03-06

**Authors:** Shuang Liang, Jing Guo, Jeong-Woo Choi, Kyung-Tae Shin, Hai-Yang Wang, Yu-Jin Jo, Nam-Hyung Kim, Xiang-Shun Cui

**Affiliations:** ^1^ Department of Animal Science, Chungbuk National University, Cheongju, Chungbuk, 361–763, Republic of Korea

**Keywords:** PP2A-B55α, oocyte maturation, cytokinesis, preimplantation development, reproduction

## Abstract

Protein phosphatase 2A regulatory subunit B55α (PP2A-B55α) has been studied in mitosis. However, its functions in mammalian meiosis and early embryonic development remain unknown. Here, we report that PP2A-B55α is critical for mouse oocyte meiosis and preimplantation embryo development. Knockdown of PP2A-B55α in oocytes led to abnormal asymmetric division, disordered spindle dynamics, defects in chromosome congression, an increase in aneuploidy, and induction of the DNA damage response. Moreover, knockdown of PP2A-B55α in fertilized mouse zygotes impaired development to the blastocyst stage. The impairment of embryonic development might have been due to induction of sustained DNA damage in embryos, which caused apoptosis and inhibited cell proliferation and outgrowth potential at the blastocyst stage. Overall, these results provide a novel insight into the role of PP2A-B55α as a novel meiotic and embryonic competence factor at the onset of life.

## INTRODUCTION

Meiosis in mammalian oocytes is a complex process during which oocytes progress from the germinal vesicle (GV) stage to the metaphase II (MII) stage upon stimulation by the luteinizing hormone surge [1]. The first indication of this process is the disappearance of GVs, as observed by light microscopy. This change is called germinal vesicle breakdown (GVBD). After GVBD, oocytes pass through the first meiotic division cycle (meiosis I) and enter the second meiotic division cycle (meiosis II). During this process, asymmetric cell division occurs after spindle migration and chromosome segregation, followed ultimately by extrusion of the small first polar body and formation of a highly polarized, large, MII-arrested oocyte that requires fertilization [2, 3]. This polarization process is critical for asymmetric cell divisions that maximize retention of the maternal cytoplasmic components required for early embryonic development [4]. Any errors in this process can lead to aneuploidy in oocytes, which is considered to be the major genetic cause of the failure of further embryonic development [4, 5]. Early reproductive events begin with folliculogenesis and end when the blastocyst implants into the uterine endometrium [6]. After fertilization, the oocyte undergoes cleavage to progress to the 2-cell, 4-cell, 8-cell, morula, and blastocyst stages, before implanting [7]. Previous research suggested that cell cycle related genes, such as JMJD3 [8], Rac1 GTPase [4], Kif1b [9], and Npm2 [10], play a vital role in successful embryonic development both before and after embryonic genome activation. Therefore, the proper regulation of these genes during oocyte maturation and early embryonic development is fundamental.

Protein phosphatase 2A (PP2A) regulatory subunit B55α (PP2A-B55α) is encoded by *Ppp2r2a* located on chromosome 8p21.2 [11]. Loss of *Ppp2r2a* inhibits homologous recombination repair through modulation of ATM phosphorylation [12]. Previous research suggested that PP2A-B55α drives p53-dependent metabolic adaptation to glutamine deprivation [13]. In addition, PP2A-B55α mediates the PP2A-Plk1 association and Plk1 dephosphorylation induced by the DNA damage response [14]. The association between PP2A-B55α and PP2A is strengthened after DNA damage in an ATM/ATR- and checkpoint kinase-dependent manner. In addition, PP2A-B55α is an important regulator of CDC25 and WEE1, which control the cell cycle [13]. PP2A-B55α antagonizes cyclin A/CDK-dependent activation of FoxM1 [15]. The FOXO1 transcription factor controls many key cellular processes, such as cell cycle arrest, cell proliferation, apoptosis, glucose and lipid metabolism, and signaling of other cellular stresses [16–18]. In pancreatic β-cells, PP2A-B55α catalyzes FOXO1 dephosphorylation under oxidative stress [18]. In addition, PP2A-B55α plays an important role in mitosis/cell cycle progression via its targets including CDK1 substrates [19]. PP2A-B55α is a critical regulator of mitotic spindle breakdown and reassembly of the nuclear envelope and Golgi apparatus during mitotic exit [19].

We hypothesized that PP2A-B55α is involved in oocyte maturation and early embryonic development. In this study, we investigated the localization, expression, and function of PP2A-B55α in oocyte meiosis and embryonic development in an attempt to expand our knowledge of its role in asymmetric division and early embryonic development. Our results show that PP2A-B55α is required for oocyte asymmetric division, spindle dynamics, and chromosome congression, as well as early embryonic development.

## RESULTS

### Localization and expression of PP2A-B55α in mouse oocytes and preimplantation embryos

**Figure 1:**
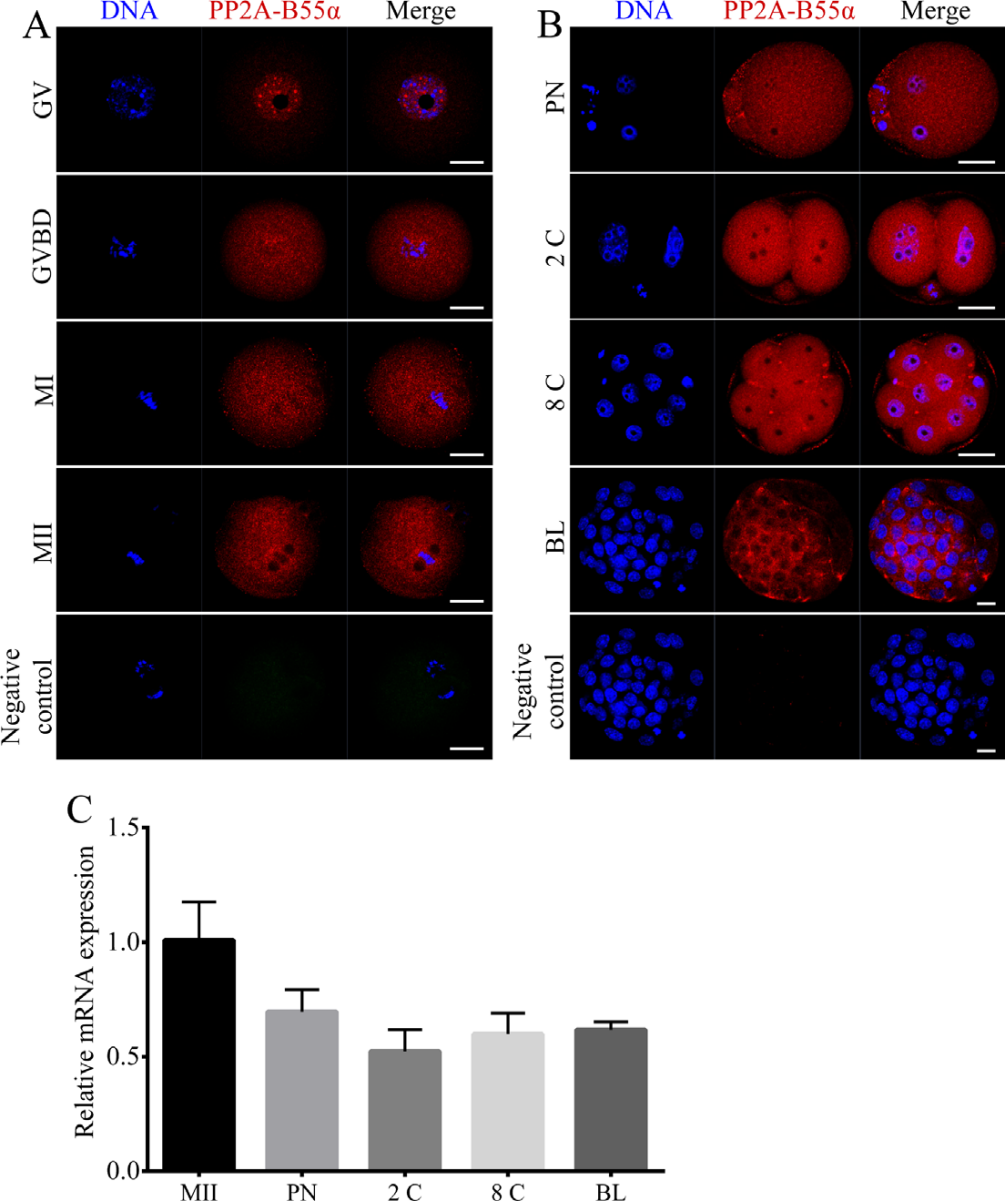
Localization and expression patterns of PP2A-B55α in mouse oocytes and preimplantation embryos (**A**) Subcellular localization of PP2A-B55α from the GV stage to the MII stage of mouse oocyte meiotic maturation. PP2A-B55α mainly localized in the nucleus at the GV stage. After the GVBD stage, PP2A-B55α was distributed throughout the oocyte. (**B**) Subcellular localization of PP2A-B55α during mouse embryonic development. Blue, DNA; red, PP2A-B55α. Bar = 20 μm. (**C**) PP2A-B55α transcript levels determined by real-time RT-PCR at different stages of mouse oocyte meiotic maturation and embryonic development. 2C: 2-cell; 8C: 8-cell; BL: blastocyst.

To investigate the expression patterns of PP2A-B55α in mouse oocytes and preimplantation embryos, we determined the subcellular localization of PP2A-B55α and temporal changes in its transcript level during mouse oocyte maturation and early embryonic development. Immunofluorescence analysis showed that PP2A-B55α was mainly localized in the nucleus at the GV stage (Figure 1A). No specific accumulation of PP2A-B55α was observed after the GVBD stage; PP2A-B55α was distributed throughout the cytoplasm, similar to its distribution in preimplantation embryos (Figure 1B). Next, PP2A-B55α transcripts were detected by quantitative RT-PCR. PP2A-B55α transcript was detected from the MII stage oocyte to the blastocyst stage (Figure 1C).

### PP2A-B55α knockdown (KD) at the GV stage does not affect oocyte maturation

**Figure 2:**
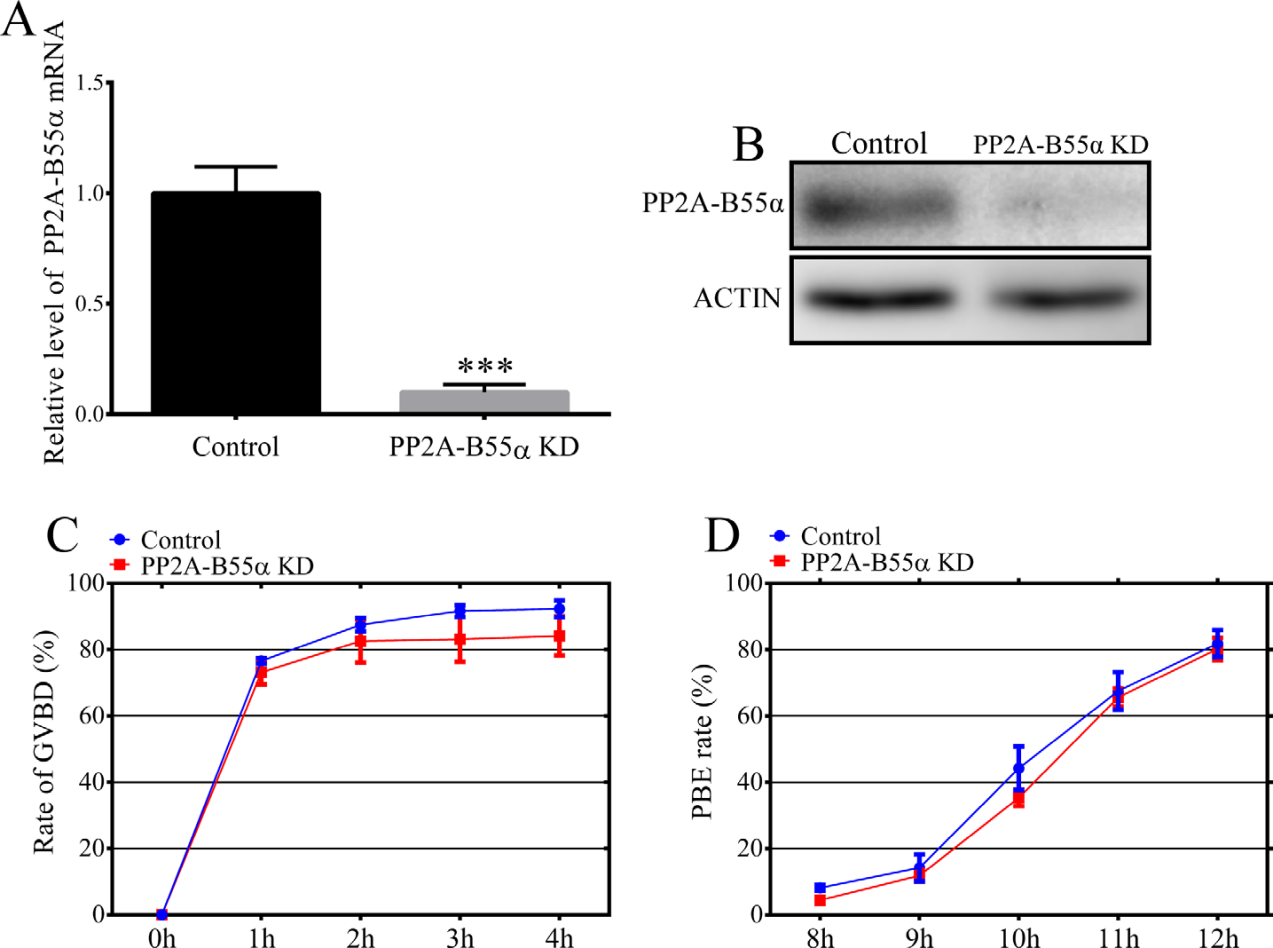
Effects of PP2A-B55α knock down on mouse oocyte maturation (**A**) Endogenous PP2A-B55α was knocked down by injecting PP2A-B55α-targeting dsRNA. The PP2A-B55α levels after dsRNA microinjection are shown and were confirmed by western blot analysis (**B**). Percentages of cultured, dsRNA-injected, GV-stage oocytes that underwent GVBD (**C**) and PBE (**D**). Meiotic resumption was investigated at 0, 1, 2, 3, and 4 h. PBE was investigated at 8, 9, 10, 11, and 12 h. The data are the mean ± SD of three independent experiments. Statistically significant differences are indicated by asterisks (***p < 0.001).

We used an RNA interference approach to investigate the functional roles of PP2A-B55α during meiotic maturation of mouse oocytes. A long double-stranded RNA (dsRNA) that specifically targeted PP2A-B55α mRNA was microinjected into GV-stage oocytes. The mRNA level of PP2A-B55α was much lower in PP2A-B55α-KD oocytes than in control oocytes (*p* < 0.001) (Figure 2A). KD of PP2A-B55α was also confirmed by western blotting (Figure 2B). Next, the effect of PP2A-B55α-KD on mouse oocyte maturation was examined. We tracked meiotic resumption and polar body extrusion (PBE) at various time points after release from milrinone, which maintains oocyte at the GV stage. Meiotic resumption, as assessed by the percentage of oocytes at the GVBD stage, and the PBE rate did not significantly differ between control and PP2A-B55α-KD oocytes (*p* > 0.05) (Figure 2C and 2D).

### PP2A-B55α-KD at the GV stage induces spindle defects, misaligned chromosomes, and aneuploidy during oocyte maturation

**Figure 3:**
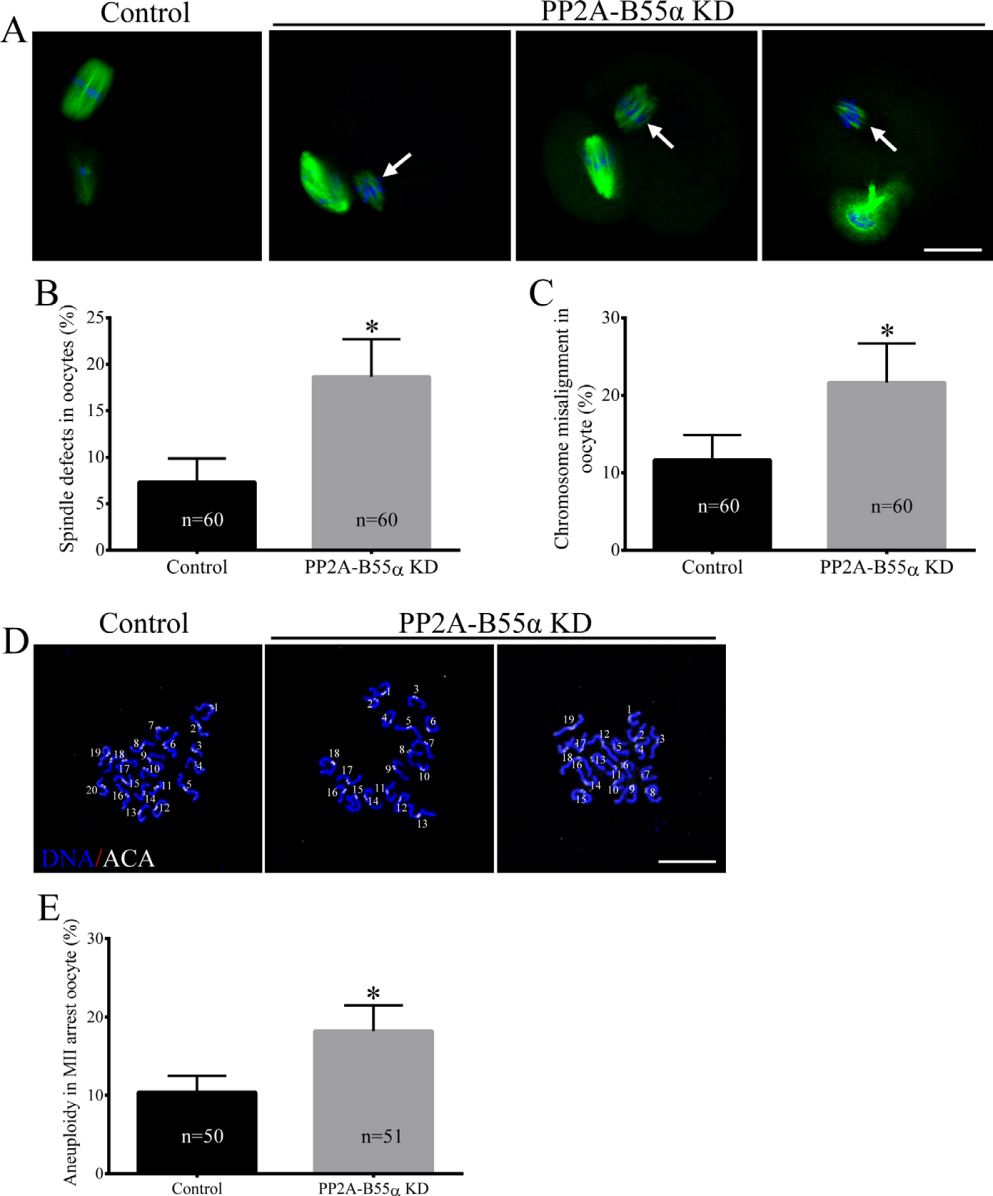
Knock down of PP2A-B55α impairs spindle assembly and chromosome alignment and increases the incidence of aneuploidy during oocyte meiosis (**A**) Representative confocal images of control and PP2A-B55α-KD oocytes at 12 h after release from milrinone are shown. Control oocytes (left) exhibited normal spindles and well-aligned chromosomes at the metaphase equator, whereas PP2A-B55α-KD oocytes displayed various spindle defects and misaligned chromosomes (arrows). Percentages of abnormal oocytes displaying aberrant spindles (**B**) and misaligned chromosomes (**C**) after PP2A-B55α-KD. Blue, DNA; green, α-tubulin. Bar = 20 μm. (**D**) Chromosome spreads (stained with Hoechst 33342, blue) and kinetochores (stained with anti-centrosome antibody [ACA], white) of MII-stage oocytes. Representative confocal images are shown. Bar = 10 μm. (**E**) Quantification of aneuploidy in control and PP2A-B55α-KD oocytes. The data are the mean ± SD of three independent experiments. Statistically significant differences are indicated by asterisks (*p < 0.05).

Next, we investigated the effects of PP2A-B55α- KD on chromosome alignment and segregation. For this purpose, control and PP2A-B55α-KD oocytes at the MII stage were immunolabeled with an anti-α-tubulin antibody to visualize the spindle and counterstained with Hoechst 33342 to label chromosomes. Significantly higher percentages of PP2A-B55α-KD oocytes than control oocytes exhibited abnormal spindles (7.3 ± 1.5% vs. 18.7 ± 2.3%, *p* < 0.05) and misaligned chromosomes (11.7 ± 1.9% vs. 21.7 ± 2.9%, *p* < 0.05) (Figure 3A–3C). PP2A-B55α- KD caused spindle defects and chromosome misalignment; therefore, we investigated the ploidy of MII-stage oocytes by preparing chromosome spreads and labeling kinetochores (Figure 3D). A significantly higher percentage of PP2A-B55α-KD oocytes than control oocytes exhibited aneuploidy (10.4 ± 0.9% vs. 18.2 ± 1.5%, *p* < 0.05; Figure 3E). These results suggest that PP2A-B55α-KD severely compromises spindle assembly and chromosome separation during oocyte maturation.

### PP2A-B55α-KD at the GV stage triggers the DNA damage response

**Figure 4:**
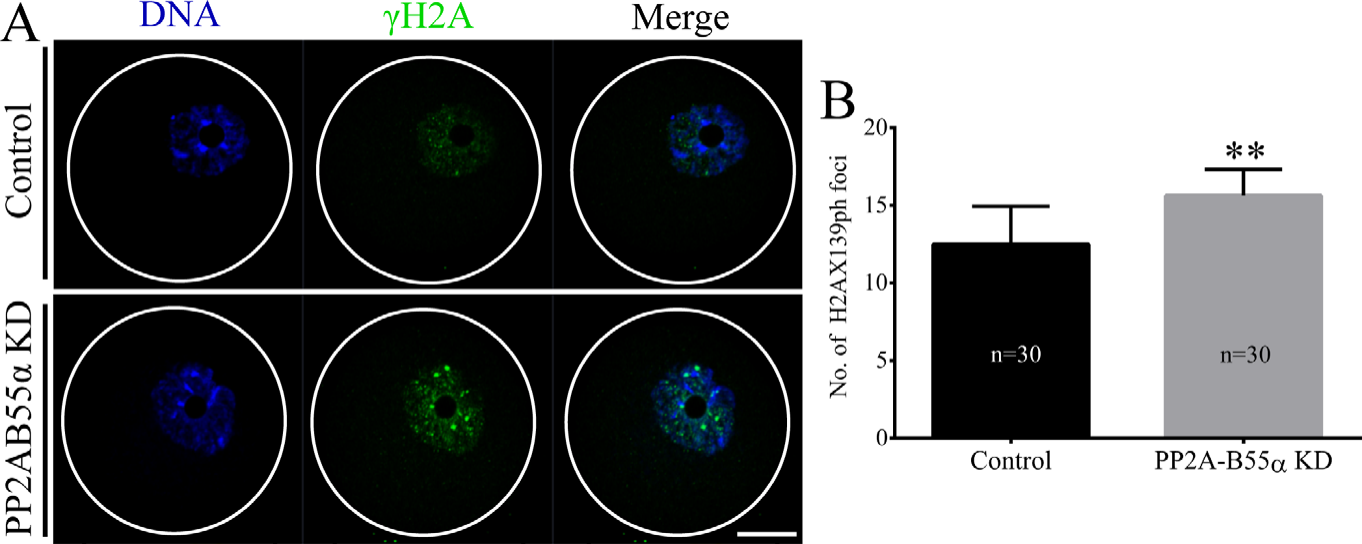
PP2A-B55α knock down triggers the DNA damage response in oocytes (**A**) Localization of γH2AX in nuclei of GV-stage oocytes. Blue, DNA; green, γH2AX. Bar = 20 μm. (**B**) Quantification of γH2AX levels in nuclei of control and PP2A-B55α-KD oocytes. The numbers of oocytes examined in each experimental group are shown in the bars. The data are mean ± SD of three independent experiments. Statistically significant differences are indicated by asterisks (**p < 0.01).

Given that PP2A-B55α is associated with DNA damage, we next analyzed DNA lesions in PP2A-B55α-KD oocytes. We performed immunofluorescence staining to visualize phosphorylated H2AX (γH2AX), a widely used marker of DNA double strand breaks (DSBs), in control and PP2A-B55α-KD oocytes (Figure 4A). γH2AX was almost completely absent in control oocytes but was abundant in the nuclei of PP2A-B55α-KD oocytes. Quantification of γH2AX showed that the γH2AX level in oocytes was greatly enhanced after PP2A-B55α-KD (*p* < 0.01; Figure 4B). These results suggest that PP2A-B55α-KD causes the formation of DNA lesions in oocytes.

### KD of PP2A-B55α reduces the developmental capacity of mouse embryos

**Figure 5:**
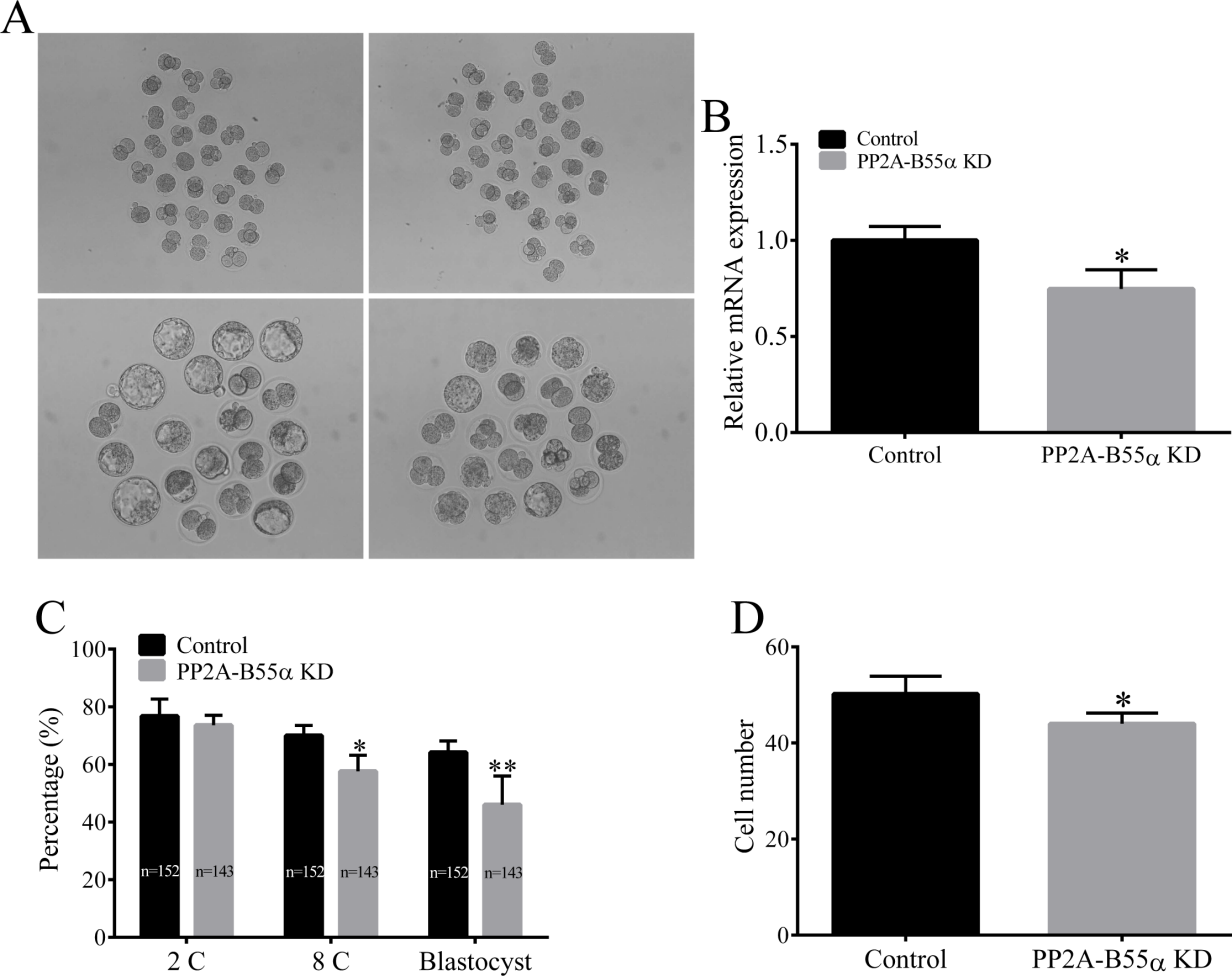
Knock down of PP2A-B55α impairs embryonic development to the blastocyst stage (**A**) Representative images of control and PP2A-B55α-KD blastocysts at 4.5 days. Bar = 50 μm. (**B**) Endogenous PP2A-B55α mRNA expression levels analyzed by real-time RT-PCR at the blastocyst stage after injection of PP2A-B55α-targeting dsRNA. Expression was normalized to that in the control group. *Ppia* was used as an internal standard because it was not affected by PP2A-B55α-targeting dsRNA injection. (**C**) Embryonic development rates in the control and PP2A-B55α-KD groups. (**D**) The number of cells in blastocysts was determined by counting cells in Hoechst 33342-stained embryos. The numbers of embryos examined in each experimental group are shown in the bars. The data are mean ± SD of three independent experiments. Statistically significant differences are indicated by asterisks (*p < 0.05; **p < 0.01).

To investigate the function of PP2A-B55α in mouse embryonic development, we microinjected PP2A-B55α-targeting dsRNA into fertilized zygotes and cultured them for 4.5 days in potassium simplex optimization medium (KSOM) to monitor their developmental potential (Figure 5A). Efficient KD of PP2A-B55α mRNA was confirmed by quantitative RT-PCR analysis of embryos that reached the blastocyst stage (*p* < 0.05; Figure 5B). Injection of fertilized zygotes with PP2A-B55α-targeting dsRNA did not affect the percentage of embryos that reached the 2-cell stage after *in vitro* culture. On the other hand, the percentages of fertilized zygotes that developed to the 8-cell and blastocyst stages were significantly decreased in the PP2A-B55α-KD group (Figure 5C). Moreover, the number of cells per blastocyst tended to be lower in the PP2A-B55α-KD group than in the control group (*p* < 0.05; Figure 5D). Collectively, these results suggest that PP2A-B55α-KD reduces the developmental capacity of mouse fertilized embryos and results in a decreased number of cells per blastocyst.

### KD of PP2A-B55α increases the DNA damage response in mouse embryos

**Figure 6:**
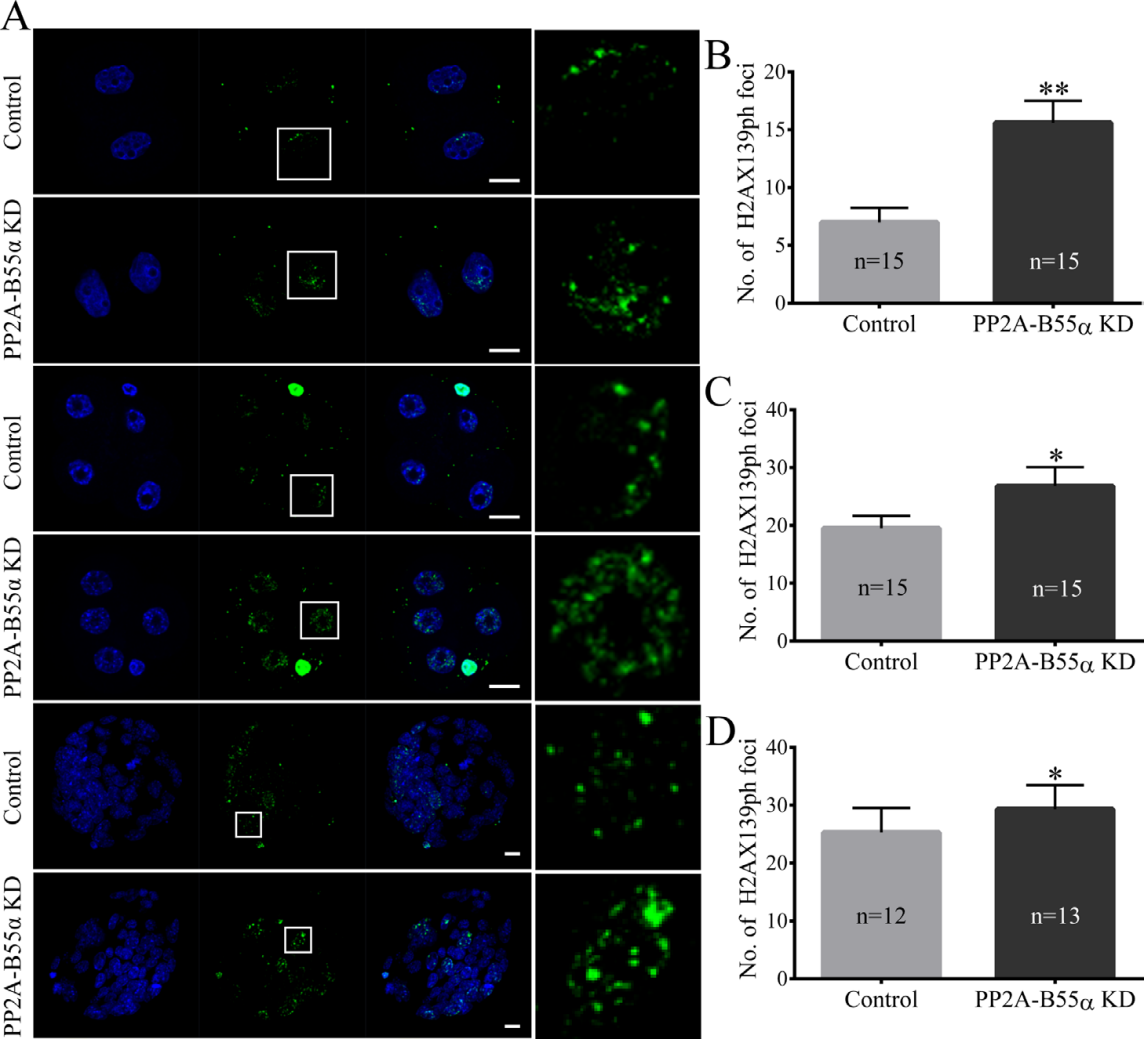
Knock down of PP2A-B55α affects the level of DNA damage in mouse embryos (**A**) Representative fluorescence images showing the presence of γH2AX139ph protein in nuclei of embryos at different developmental stages. Nuclei in embryos were stained blue and γH2AX139ph foci were stained green. Bar = 20 μm. The average numbers of γH2AX139ph foci in nuclei of PP2A-B55α-KD embryos are shown at the 2-cell (**B**), 4-cell (**C**), and blastocyst (**D**) stages. The numbers of embryos examined in each experimental group are shown in the bars. The data are the mean ± SD of three independent experiments. Statistically significant differences are indicated by asterisks (*p < 0.05, **p < 0.01).

To explore the mechanism by which PP2A-B55α-KD reduces the developmental capacity of fertilized mouse embryos, we examined γH2AX in mouse embryos at various stages of development. The nuclear γH2AX level was higher in PP2A-B55α-KD embryos than in control embryos (Figure 6). These results suggest that DNA DSBs accumulate in fertilized embryos after PP2A-B55α disruption.

### KD of PP2A-B55α increases apoptosis and reduces cell proliferation in mouse embryos

**Figure 7:**
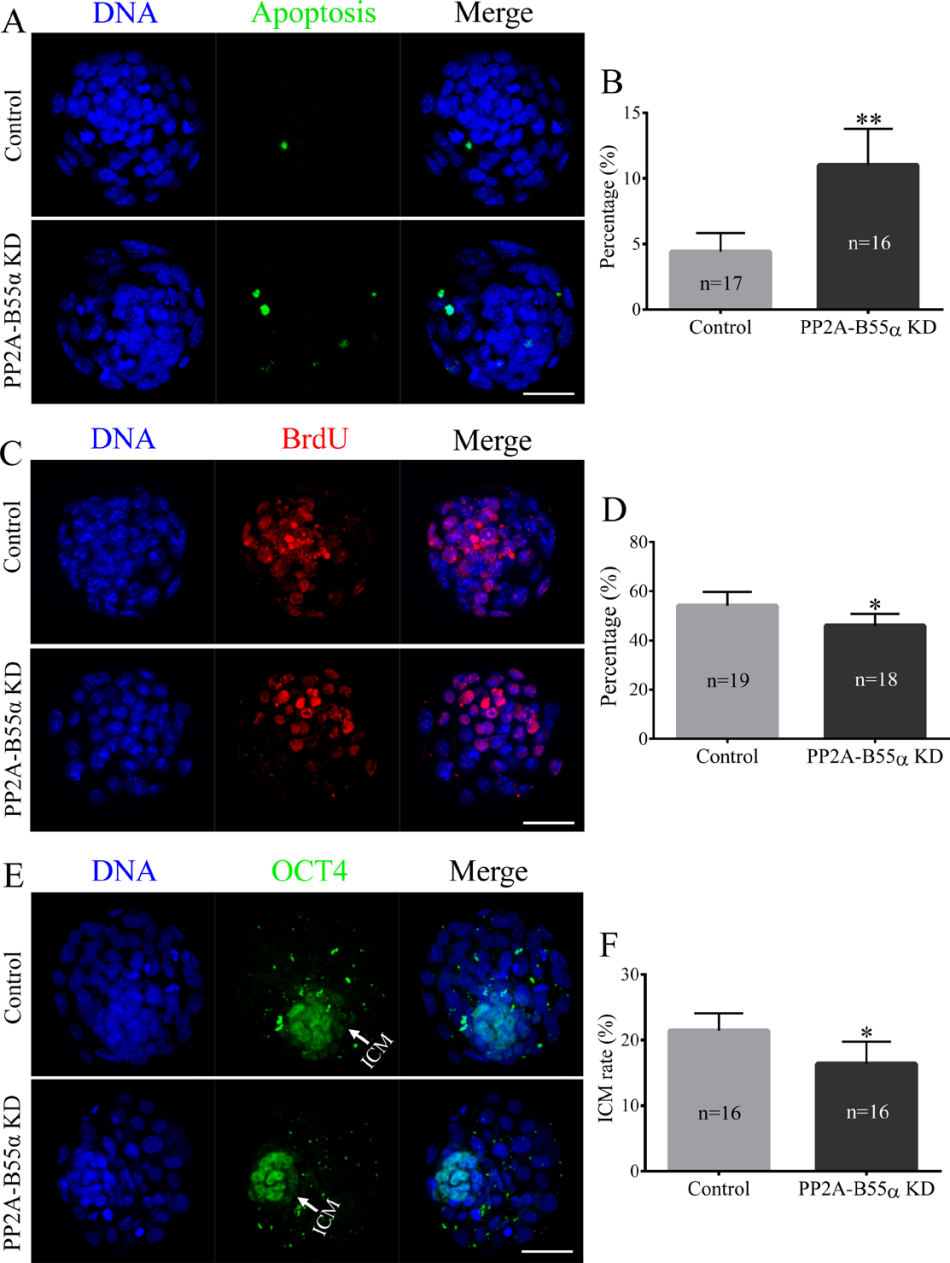
Knock down of PP2A-B55α affects the levels of apoptosis and cell proliferation in mouse embryos (**A**) Representative images of embryos at the blastocyst stage in the TUNEL assay. Bar = 50 μm. (**B**) The percentage of apoptotic cells in blastocysts that developed *in vitro*. (**C**) Immunofluorescence staining of BrdU in mouse embryos at the blastocyst stage. Bar = 50 μm. (**D**) Percentages of BrdU-positive cells in blastocysts. (**E**) Immunofluorescence staining of OCT4 in mouse embryos at the blastocyst stage. Bar = 50 μm. (**F**) ICM rate of blastocysts. The numbers of blastocysts examined in each experimental group are shown in the bars. The data are the mean ± SD of three independent experiments. Statistically significant differences are indicated by asterisks (*p < 0.05, **p < 0.01).

**Figure 8:**
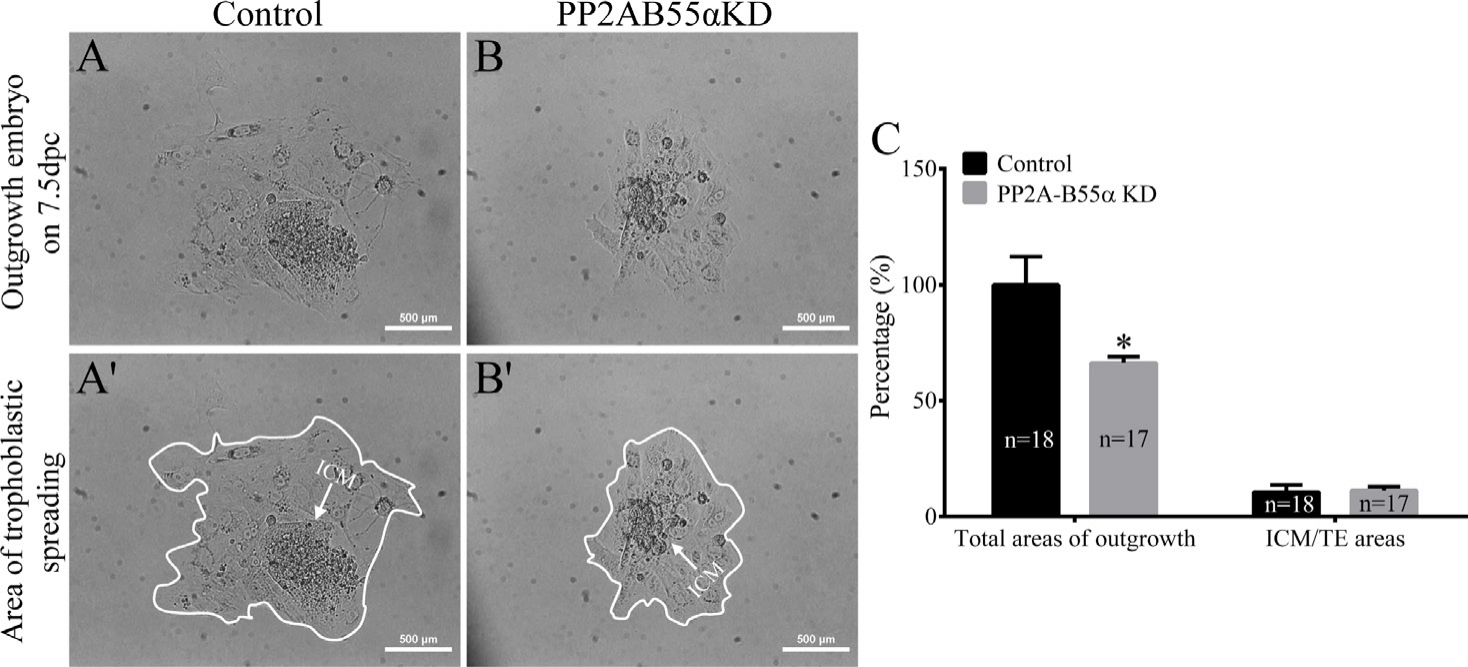
Knock down of PP2A-B55α affects the implantation potential of mouse embryos (**A**) Representative images showing blastocyst outgrowth at 7.5 dpc in the control (A and A') and PP2A-B55α-KD (**B** and B') groups. Bar = 100 μm. (**C**) The total areas of outgrowth and ICM/TE areas in the control and PP2A-B55α-KD groups. The numbers of blastocysts examined in each experimental group are shown in the bars. The data are the mean ± SD of three independent experiments. Statistically significant differences are indicated by asterisks (*p < 0.05).

Apoptosis occurs in response to excessive DNA DSBs in cells. Apoptotic cells were detected in blastocysts using the TUNEL assay (Figure 7A). The percentage of apoptotic cells was significantly higher in PP2A-B55α-KD blastocysts than in control blastocysts (*p* < 0.01; Figure 7B). This result suggests that PP2A-B55α plays an important role in regulation of apoptosis during mouse embryonic development. We also evaluated the proliferative capacity using the 5-bromodeoxyuridine (BrdU) assay. Representative images of proliferating cells in blastocysts are shown in Figure 7C. The percentage of cells exhibiting DNA synthesis was significantly lower in PP2A-B55α-KD blastocysts than in control blastocysts (*p* < 0.05; Figure 7D). In addition, the ICM rate was lower in the PP2A-B55α-KD group than in the control group (*p* < 0.05; Figure 7E and 7F). To further investigate the influence of PP2A-B55α-KD on blastocysts, the ability of embryos to implant was assessed *in vitro*. Blastocysts were followed daily until day 7.5 of development to evaluate their implantation potential *in vitro* by measuring the trophoblastic spreading area by an outgrowth assay. The total area of outgrowth was significantly smaller in the PP2A-B55α-KD group than in the control group (*p* < 0.05; Figure 8A and 8B). These findings demonstrate that PP2A-B55α-KD impairs the developmental potential of mouse preimplantation embryos.

## DISCUSSION

Mammalian oocyte maturation is a complex process that involves asymmetric cell division to ultimately form a highly polarized, large, MII-arrested oocyte that requires fertilization. This polarization process is critical for fertilization and early embryonic development. Any errors in this process can impair oocyte maturation and further embryonic development. Here, we demonstrated an essential role of PP2A-B55α in mouse oocyte meiotic maturation and early embryonic development. Furthermore, the results demonstrated that downregulation of PP2A-B55α in mouse oocytes and embryos impairs oocyte asymmetric division and early embryonic development.

We first examined whether PP2A-B55α was expressed in mouse oocytes and early embryos by performing immunofluorescence labeling. PP2A-B55α exhibited specific expressions and localizations in both oocytes and embryos. PP2A-B55α was mainly localized in the nucleus at the GV stage. After the GVBD stage, PP2A-B55α was distributed throughout the cytoplasm, similar to its distribution in preimplantation embryos. The multiple isoforms of the regulatory B subunits give rise to the diversity of PP2A holoenzymes [18, 20]. Previous studies have found that the subcellular localization of PP2A holoenzyme was controlled by the variable B subunits [21, 22]. In the PP2A subfamily, the individual B subunits are specific to tissue and cell type or developmental stage [21–24]. Thus, this specific expression pattern indicates that PP2A-B55α is involved in oocyte meiotic maturation and early embryonic development.

Meiotic spindles are important for chromosome alignment and separation of maternal chromosomes during fertilization. During meiotic maturation, the meiotic spindle assembles around the centrally positioned metaphase chromosomes and then migrates to the cortex of the oocyte. Errors at any step in this process can lead to abnormal chromosome segregation and resultant aneuploidy in oocytes [25]. PP2A-B55α is associated with the DNA damage response during mitosis [26]. Specifically, PP2A-B55α attaches telomeres of chromosomes to the nuclear envelope, and depletion of PP2A-B55α protein triggers the DNA damage response. In the present study, PP2A-B55α-KD mouse oocytes exhibited up-regulation of γH2AX, indicative of the DNA damage response, at the GV stage (Figure 4). Thus, downregulation of PP2A-B55α might cause abnormal asymmetric meiotic division. Collectively, on the basis of these data, we conclude that PP2A-B55α is required for orderly meiosis in mammalian oocytes.

To further explore why abnormal asymmetric division occurred in PP2A-B55α-KD oocytes, we investigated spindle organization and chromosome alignment in MI-stage oocytes. Unexpectedly, spindle organization and chromosome alignment were not affected in MI-stage oocytes (Supplementary Figure 1). This result differs from previous studies, which reported that PP2A is involved in MI spindle formation and suggested that PP2A-B55α is not required for microtubule polymerization at MI stage [27, 28]. In addition, anaphase-promoting complex/cyclosome (APC/C) activity was also detected. APC/C is one of ubiquitin ligases, which plays essential functions during cell cycle progression [29]. The activity of the APC/C is essential for spindle disassembly and spindle-pole separation. APC/C promotes degradation of cyclin B and securin, thereby permitting chromosome segregation and PBE [30]. In addition, degradation of cyclin B is required for inhibition of CDK1 activity followed by the disassembly of the mitotic spindle and cytokinesis [29]. Time-lapse live-cell imaging was performed of oocytes injected with mRNA encoding GFP-tagged cyclin B, which determines APC/C activity. We found that PP2A-B55α did not affect cyclin B degradation in oocytes (Supplementary Figure 2A and 2B). APC/C activity is also activated by the spindle assembly checkpoint (SAC) [31]. SAC proteins play critical roles in precisely monitoring chromosome segregation [32]. To confirm this phenotype, we further determined the level of the SAC protein Bub3. The Bub3 level at kinetochores was not decreased in PP2A-B55α-KD oocytes (Supplementary Figure 2C and 2D). These results suggest that PP2A-B55α is not associated with APC/C activity.

We next investigated the function of PP2A-B55α during mouse preimplantation embryo development. We found that PP2A-B55α plays a critical role in early mouse development (Figure 6). Following KD of PP2A-B55α, lower percentages of zygotes developed to the 8-cell and blastocyst stages, and the blastocysts contained fewer cells. However, there was no effect on the percentage of embryos that reached the 2-cell stage, suggesting that PP2A-B55α activity is not required for the initial cell divisions in mouse embryos. These data highlight the importance of PP2A-B55α in the regulation of mouse early embryonic development.

PP2A-B55α plays a direct or indirect role in DNA DSB repair by activating several proteins involved in repair pathways [14, 26]. Depletion of PP2A-B55α significantly increases AKT Thr-308 phosphorylation [33]. In addition, PP2A-B55α increases phosphorylation of ATM and activation of CHK2 kinase, leading to cell cycle arrest [12]. The results of immunofluorescence staining showed that PP2A-B55α was mainly localized cytoplasmic during mouse early embryonic development. It is similar with mitochondria localization pattern, together with that mitochondria is closed to DNA damage. These findings led us to investigate the effects of PP2A-B55α on DNA DSBs in mouse embryos at different developmental stages. KD of PP2A-B55α induced the accumulation of DNA DSBs during early embryonic development, as determined by analyzing the level of γH2AX, which is commonly used as a biomarker of the cellular response to DNA DSBs and to monitor DNA damage and repair [34]. Therefore, PP2A-B55α-KD may arrest embryonic development via increasing the accumulation of DNA damage during early embryonic development.

DNA damage not only arrests the cell cycle, but can also lead to activation of apoptotic pathways. Apoptosis was enhanced after PP2A-B55α-KD, which led us to speculate that PP2A-B55α may play a role in the execution of apoptosis. In addition, KD of PP2A-B55α decreased cell proliferation and the ICM rate in blastocysts. These results support the notion that downregulation of PP2A-B55α impairs the developmental competence of mouse preimplantation embryos. Successful blastocyst implantation is essential for reproduction. Embryonic outgrowth is usually used as an *in vitro* model to study peri-implantation embryonic development and the process of trophoblastic invasion during implantation of embryos into the uterine wall [35, 36]. Outgrowth of trophoblast cells from cultured blastocysts is believed to reflect the proper differentiation of embryos [35]. In the present study, we found that the *in vitro* implantation potential of blastocysts was significantly decreased after PP2A-B55α-KD. These results strongly support our hypothesis that KD of PP2A-B55α negatively affects mouse early embryonic development.

In conclusion, the present study demonstrated that KD of PP2A-B55α impairs mouse oocyte meiotic maturation and early embryonic development, and decreases the quality of embryos. Further studies are needed to further understand the mechanism underlying regulation of mouse oocyte meiotic maturation and embryonic development by PP2A-B55α.

## MATERIALS AND METHODS

### Animals and chemicals

The experimental protocol was approved by the Ethics Committee of Chungbuk National University. Native-breed female ICR mice were used in all experiments. Mice were kept in a temperature-controlled room (22–25°C) with a dark/light cycle of 12 h of light and 12 h of darkness, adequate ventilation, hygienic conditions, and free access to a regular diet. All chemicals used in this study were purchased from Sigma Chemical Company (St. Louis, MO, USA), unless otherwise noted.

### Oocyte and zygote collection

Female ICR mice (6−8-weeks-old with body weights of 25–30 g) were sacrificed by cervical dislocation. Ovaries were isolated and cut using a blade to release immature oocytes. Only oocytes with an intact GV were collected for further studies. Immature oocytes were isolated in M2 medium containing 2.5 mM milrinone to maintain them at the GV stage during microinjection.

To obtain zygotes, female ICR mice were superovulated via an intraperitoneal injection of 10 IU of pregnant mare serum gonadotrophin, followed 48 h later by an intraperitoneal injection of 10 IU of human chorionic gonadotrophin (hCG). Superovulated females were mated with ICR males. Approximately 19–21 h after hCG injection, female mice were sacrificed by cervical dislocation and zygotes were collected by puncturing the ampullae of the oviducts. Cumulus cells were removed by incubation in pre-warmed M2 medium containing 0.1 mg/mL hyaluronidase for 3 min. These fertilized embryos were then washed thrice in pre-warmed M2 medium and cultured in pre-warmed M2 medium for at least 30 min to allow recovery before microinjection. Denuded zygotes with a clearly visible PN, a second polar body, and a normal morphology were selected for further experiments.

### Preparation of dsRNA and cRNA

PP2A-B55α-targeting dsRNA was prepared as previously described [5]. Briefly, a 613 bp DNA fragment of PP2A-B55α cDNA was amplified using gene-specific primers containing the T7 promoter sequence (GAATTAATACGACTCACTATAGGGAGA) at both 5′ ends. PP2A-B55α was amplified using cDNA and the PP2A-B55α-specific primer set listed in Supplementary Table S1. dsRNA was then synthesized via *in vitro* transcription at 37°C for 4 h using the purified PCR amplicons and a MEGAscript T7 Kit (Ambion, Austin, TX, USA). The synthesized dsRNA was treated with DNase I to remove any contaminating DNA and purified using phenol-chloroform extraction and isopropyl alcohol precipitation. dsRNA was stored at −80°C until microinjection.

The pRN3-cyclin B-GFP plasmids [37] were kindly provided by Dr. JungSoo Oh (Sung Kyun Kwan University, Suwon, Korea). Cyclin B-GFP were generated using a mMessage mMachine SP6 Kit (Life Technologies, Carlsbad, CA, USA). *In vitro* transcripts were purified using phenol-chloroform extraction and isopropyl alcohol precipitation, and stored at −80°C until use.

### Microinjection

Microinjection was performed using an inverted microscope (Nikon Corporation, Tokyo, Japan) and an Eppendorf FemtoJet microinjector (Eppendorf, Hamburg, Germany), and was completed within 1 h. To KD PP2A-B55α in oocytes, 3–5 pL of dsRNA (1 μg/μL) prepared in RNase-free H_2_O was microinjected into the cytoplasm of a fully grown GV-stage oocyte. Immediately after microinjection, the oocytes were washed and cultured in M16 medium containing 2.5 μM milrinone for 20 h to ensure that the targeted protein was depleted. After 20 h, the oocytes were washed thrice, transferred to fresh M16 medium, and cultured for a further 12 h under liquid paraffin oil at 37°C in a 5% CO_2_ incubator until they reached the GV (0 h), GVBD (2 h), metaphase I (MI, 6 h), and MII (12 h) stages. To KD PP2A-B55α in zygotes, 3–5 pL of dsRNA (1 μg/μL) prepared in RNase-free H_2_O was microinjected into the cytoplasm. After microinjection, the zygotes were washed thrice with KSOM and incubated in KSOM under paraffin oil in 4-well dishes at 37°C in 5% CO_2_ and air, without changing the medium. Blastocysts were collected after 4.5 days for further experiments. As the control, 3–5 pL of water was microinjected into GV-stage oocytes and zygotes under the same conditions.

### Immunofluorescence staining

Samples were fixed in 3.7% (w/v) paraformaldehyde for 30 min and transferred to membrane permeabilization solution (0.2% Triton X-100 prepared in PBS-PVA) for 1 h. After being blocked for 30 min in 1.5% bovine serum albumin prepared in PBS-PVA (blocking solution), the samples were incubated with the primary antibody diluted in blocking solution overnight at 4°C. Antibodies used to detect PP2A-B55α (1:100; Cat: #4953) and γH2AX (pS139, 1:100; Cat: #2577) were purchased from Cell Signaling Technology (USA). An anti-OCT4 antibody (1:100; Cat: sc-8628) was purchased from Santa Cruz Biotechnology (USA). After extensive washes with PBS-T (PBS containing 0.1% Tween 20 and 0.01% Triton X-100), the embryos were incubated with a fluorescein isothiocyanate (FITC)- or Texas Red-conjugated anti-rabbit IgG (secondary) antibody (Life Technologies) diluted 1:200 in PBS-T for 1 h. To stain spindles, oocytes were incubated overnight at 4°C with a FITC-conjugated anti-α-tubulin antibody diluted 1:100 in PBS-PVA. Thereafter, the embryos were washed thrice with PBS-T and incubated for 15 min with 10 μg/mL Hoechst 33342 prepared in PBS-PVA. Finally, the oocytes and embryos were mounted onto glass slides and examined using a confocal laser scanning microscope (Zeiss LSM 510 and 710 META, Oberkochen, Germany). The fluorescence intensities of PP2A-B55α were quantified using ImageJ software [38]. The number of γH2AX139ph foci was evaluated using Zeiss software. Foci larger than 0.3 μm^3^ were counted as sites of DNA DSBs [39, 40].

### Western blotting analysis

For western blotting, 200 oocytes were collected in SDS sample buffer and heated for 5 min at 95°C. Proteins were separated by SDS-PAGE and electrically transferred to polyvinylidene fluoride membranes. Membranes were blocked in TBST (Tris-buffered saline containing Tween 20) containing 5% BSA for 2 h and then incubated overnight at 4°C with the primary antibody (1:500). After washing three times in TBST (each for 10 min), membranes were incubated for 1 h at 37°C with a peroxidase-conjugated secondary antibody (1:2,000; Santa Cruz, CA, USA). Finally, membranes were processed using SuperSignal West Femto Maximum Sensitivity Substrate (Thermo Scientific, Waltham, MA, USA).

### Chromosome spreading

Chromosome spreading was performed as previously described [25, 41]. Briefly, oocytes were exposed to 1 mg/ml pronase to remove the zona pellucida and then fixed in 1% paraformaldehyde prepared in distilled water (pH 9.2) containing 0.15% Triton X-100 and 3 mM dithiothreitol. The samples were dried slowly at room temperature for several hours and then placed in blocking solution for 1 h. Antibodies used to detect kinetochores (1:50; anti-centromere; gift of Dr. Qing-Yuan Sun; Institute of Zoology, Chinese Academy of Sciences). An anti-Bub3 antibody (1:50; Cat: sc-28258) was purchased from Santa Cruz Biotechnology (USA). Chromosomes were co-stained with Hoechst 33342. A laser scanning confocal microscope was used to determine the number of chromosomes in oocytes. An abnormal number of chromosomes in oocytes (more or less than 20) was defined as aneuploidy.

### Time-lapse microscopy

Cyclin B-GFP mRNA was microinjected into GV stage oocytes, as described above. Time-lapse imaging was performed using a Lumascope 620 inverted microscope (Etaluma Inc., Carlsbad, CA, USA) installed inside an incubator maintained at 37°C and 5% CO2. Images were automatically captured every 15 min for 12 h.

### TUNEL assay and cell counting

Blastocysts were washed thrice with PBS-PVA and then fixed with 3.7% paraformaldehyde prepared in PBS-PVA for 30 min. Thereafter, blastocysts were washed with PBS-PVA and permeabilized by incubation with 0.2% Triton X-100 for 1 h. Next, the blastocysts were washed thrice with PBS-PVA and incubated with fluorescein-conjugated dUTP and the terminal deoxynucleotidyl transferase enzyme (*In Situ* Cell Death Detection Kit, Roche; Mannheim, Germany) in the dark for 1 h at 37°C. After being incubated with 10 μg/ml Hoechst 33342 for 10 min at 37°C to label all nuclei, the blastocysts were washed with PBS-PVA, mounted with slight coverslip compression, and examined under a laser scanning confocal microscope. The cells and apoptotic nuclei were counted after images were acquired.

### Proliferation analysis

The rate of cell proliferation was assessed by performing a BrdU assay as described previously with slight modifications [42]. Briefly, blastocysts were incubated with 100 mM BrdU in a humidified atmosphere of 5% CO_2_ at 38.5°C for 6 h. The blastocysts were then washed with PBS containing 0.05% Tween 20 (PBS-T), fixed in ice-cold methanol for 20 min, and permeabilized at room temperature with 0.2% Triton X-100. Thereafter, the blastocysts were washed with PBS-T and treated with 2 N HCl at room temperature for 30 min. Next, the blastocysts were washed and incubated with a mouse anti-BrdU monoclonal antibody (Cat: B2531) diluted 1:10 at 4°C overnight. After a wash in 0.1% bovine serum albumin prepared in PBS, the blastocysts were incubated with a rabbit anti-mouse IgG Alexa Fluor 568-conjugated polyclonal antibody (Cat: A11061, diluted 1:100) at room temperature for 1 h. After extensive washes with PBS-T, embryos were counterstained with 10 μg/ml Hoechst 33342, mounted on glass slides, and examined under a confocal laser scanning microscope. Proliferating cells were counted using ImageJ software.

### Blastocyst outgrowth assay

The blastocyst outgrowth assay was performed as previously described [43], with slight modifications. Briefly, the zona pellucida of blastocysts was removed using 0.25% pronase (Sigma). Zona pellucida-free embryos were cultured in Dulbecco's modified Eagle's medium containing 10% FBS without leukemia inhibitory factor. Outgrowing embryos were imaged daily with an inverted microscope and analyzed using Image J software. The area of trophoblastic outgrowth was measured on 7.5 days postcoitum (dpc) using Image J software.

### Real-time RT-PCR analysis

Total RNA was extracted from oocytes and embryos using a Dynabeads mRNA Direct Kit (Invitrogen, Grand Island, NY, USA) according to the manufacturer's instructions. First-strand cDNA was synthesized by reverse transcription of mRNA using the Oligo (dT) 12–18 primer and SuperScript TM III reverse transcriptase (Invitrogen). Real-time RT-PCR was performed with SYBR Green, a fluorophore that binds to double-stranded DNA, in a final reaction volume of 20 μl using a CFX96 touch real-time RT-PCR detection system (Bio-Rad, Hercules, CA, USA). Finally, gene expression was quantified using the 2^−ΔΔCt^ method, with normalization to the mRNA expression of *Ppia*, which usually used as housekeeping gene in mouse [44]. The PCR primers used to amplify each gene are listed in Supplementary Table 1.

### Statistical analysis

All percentage data were subjected to arcsine transformation prior to statistical analysis and presented as mean ± S.D. Comparisons of data between groups were performed with Student's *t* test. All statistical analysis was performed using the software package GraphPad Prism (version 6.01; La Jolla, CA, USA). *P* < 0.05 was considered statistically significant.

## SUPPLEMENTARY MATERIALS FIGURES AND TABLE


