# Supplementary material for: Protein phosphatase 2A regulatory subunit B55α functions in mouse oocyte maturation and early embryonic development
Source: Oncotarget. 2017 Mar 6;8(16):26979–91. doi: 10.18632/oncotarget.15927 (PMC5432312; doi:10.18632/oncotarget.15927)
Supplement: Supplementary file 1 [file oncotarget-08-26979-s001.pdf]

Protein phosphatase 2A regulatory subunit B55α functions in mouse oocyte maturation and early embryonic development

Supplementary Materials

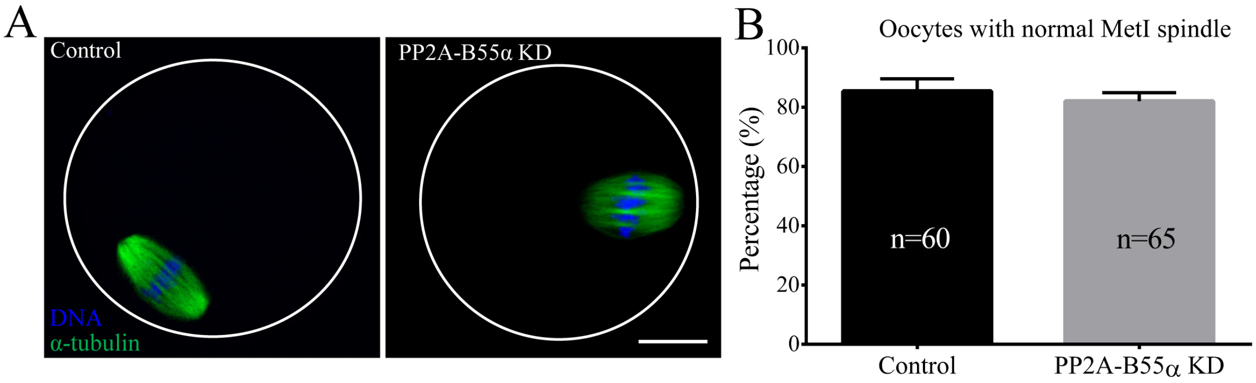

Supplementary Figure 1: PP2A-B55α knock down does not affect spindle formation at the MI stage. (**A**) Oocytes at the MI stage were stained with an anti-α-tubulin antibody. (**B**) Percentage of oocytes with a normal spindle at the MI stage. Blue, DNA; green, α-tubulin. Bar = 20 µm. The data are the mean ± SD of three independent experiments.

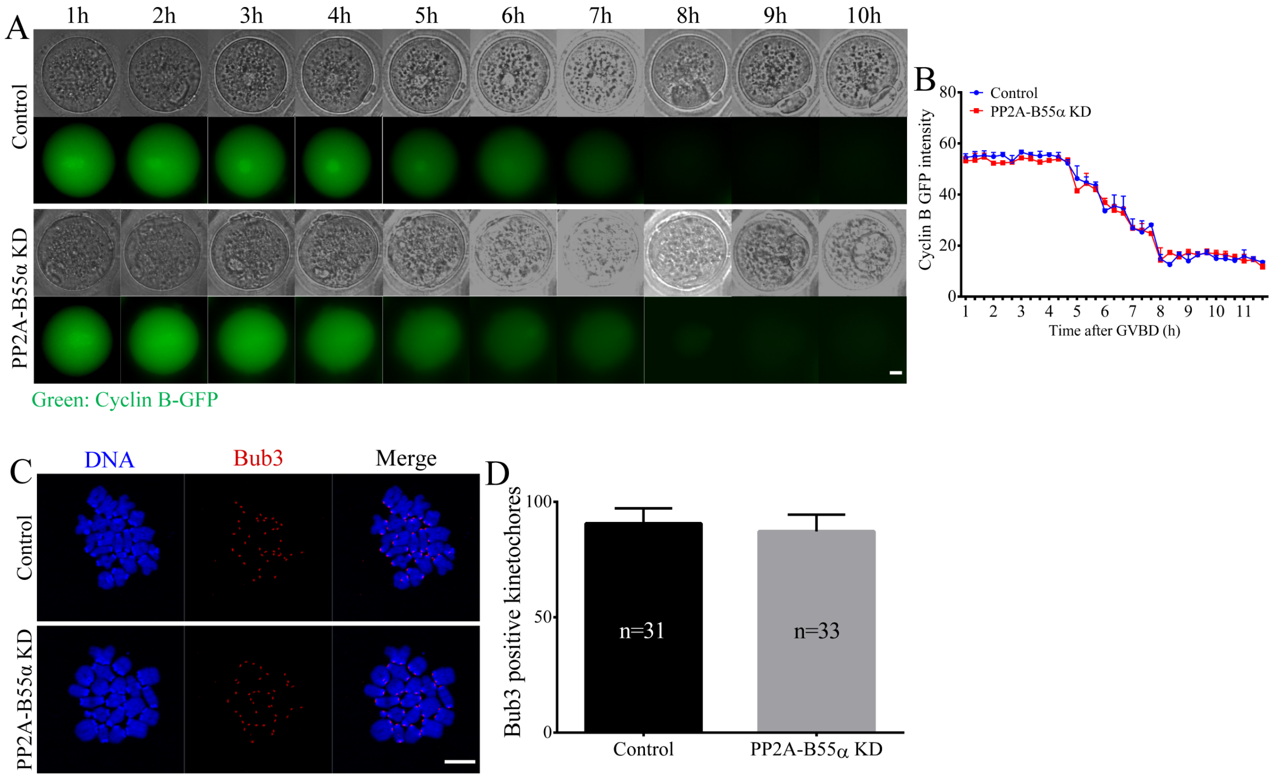

Supplementary Figure 2: SAC activity in PP2A-B55α knock down oocytes. (**A**) Time-lapse live-cell imaging of PP2A-B55α-KD oocytes expressing cyclin B-GFP mRNA. Time points indicate the culture duration. GFP levels were measured every 15 min. (**B**) Quantification of cyclin B-GFP levels. Green: cyclin B-GFP. Bar = 20 µm. (**C**) Oocytes at the MI stage were stained with a Bub3 antibody. (**D**) Percentage of Bub3 positive kinetochores in the MI stage oocyte. Blue, DNA; red, Bub3. Bar = 20 µm. The data are the mean ± SD of three independent experiments.

Supplementary Table 1: Primers used in this study

Gene	Primer sequence	Use of the primer
PP2A-B55α	F: 5′GAATTAATACGACTCACTATAGGGAGAAGTGCCCGTATTTAGGCC3′	dsRNA
	R: 5′GAATTAATACGACTCACTATAGGGAGAACTGTGCCTGTCAGACCC3′	
	F: 5′AGAGGAAGATGGAAGATATAGAG3′	qPCR
	R: 5′GAGTTGATGTGATAAGTGTGG3′	
Ppia	F: 5′CGCGTCTCCTTCGAGCTGTT3′	qPCR
	R: 5′TGTAAAGTCACCACCCTGGC3′	

F: forward; R: reverse; dsRNA: double-stranded RNA; qPCR: quantitative PCR.
